# Comparing deep learning stroke segmentation in NCCT, CTA, and CTP: Accuracy, domain transfer, and temporal sampling effect

**DOI:** 10.1002/mp.70419

**Published:** 2026-04-03

**Authors:** Linda Vorberg, Hendrik Ditt, Andreas Maier, Savvas Nicolaou, Nicolas Murray, Oliver Taubmann

**Affiliations:** ^1^ Pattern Recognition Lab Friedrich‐Alexander Universität Erlangen‐Nürnberg Erlangen Germany; ^2^ Computed Tomography Siemens Healthineers AG Forchheim Germany; ^3^ Department of Radiology Vancouver General Hospital University of British Columbia Vancouver Canada

**Keywords:** computed tomography, segmentation, stroke

## Abstract

**Background:**

Stroke imaging typically involves multiple CT image types—non‐contrast CT (NCCT), CT angiography (CTA), and CT perfusion (CTP). CTP and multiphase CTA (mCTA) are more advanced acquisitions with multiple timesteps and provide insights on the hemodynamics within the brain. Deep Learning models can help facilitate the diagnostic workflow by automatically identifying the extent of core and penumbra, which influences subsequent treatment decisions. For the use in clinical practice, generalizability of these models to new clinical sites is crucial.

**Purpose:**

We evaluate and compare the usefulness of NCCT, CTA, mCTA, and CTP images for DL‐based stroke lesion segmentation, with the aim of guiding modality selection in settings with and without access to advanced imaging, and with an additional focus on model transferability between clinical sites and the impact of time point selection from the CTP scan.

**Methods:**

The experiments involve model training with a dataset of 91 stroke patients from one clinical site. NCCT, CTA, mCTA, and CTP are used separately to train nnU‐Net models for segmentation of stroke core and hypoperfused volume using uncertainty‐aware labels. To assess site transferability, a model (pre‐)trained on 166 cases from a second clinical site is employed to perform as‐is inference with data from the first site, then contrast it with a variant of the model fine‐tuned using a subset of the data from the first site. Multiple temporal sampling strategies were investigated for the 4D CTP data, choosing different subsets of the time series as the model input.

**Results:**

For automatic segmentation of stroke core, advanced imaging techniques yield improved accuracy with the modified Dice coefficient increasing from 0.36±0.28 (NCCT) to 0.55±0.27 (CTA), 0.71±0.22 (mCTA), and 0.78±0.09 (CTP) for infarcts of size 10–70  mL. A similar trend is observed for smaller infarcts of 1–10 mL. In terms of generalizability, the additional fine‐tuning stage consistently enhances the segmentation results, regardless of the image type used. To leverage the initially large series of perfusion images, different temporal sampling strategies are applied to predict stroke core. The experiments show no clear trend as the results vary across different timing scenarios and infarct sizes.

**Conclusions:**

The study provides an overview of the quality of automated stroke lesion segmentation with nnU‐Net across all relevant CT acquisition types. Hereby, multitimepoint imaging exhibits significantly improved segmentation performance compared to NCCT and CTA.

## 1 INTRODUCTION

Stroke is one of the leading causes of death and disability world wide with the majority of cases (62.4%) of the ischemic type, meaning that they are caused by a blockage or vessel occlusion while another 10% of cases are hemorrhagic due to the rupture of a blood vessel.^1^ In the diagnostic workflow of acute ischemic stroke, CT imaging plays a vital role due to its widespread availability and rapid acquisition, as a timely diagnosis is crucial for further treatment. Typically, the first image to be acquired is a noncontrast CT (NCCT) scan to exclude hemorrhage. In some cases, early ischemic changes can already be visible in these scans but may be subtle and depend on the time since stroke onset.^2^ After the initial scan, CT angiography (CTA) with the administration of contrast agent follows to visualize the brain vasculature and detect the location of the vessel occlusion. A closely related technique is the multiphase CTA (mCTA) scan, which includes the acquisition of several (mostly two) phases after the arrival of contrast agent, in addition to the initial CTA scan.^3^ This is done to assess the collateral status, detect smaller vessel occlusions, and determine thrombus length but can also be used to identify perfusion deficits in stroke patients.^4^ A more advanced scan with contrast agent is CT perfusion (CTP), which involves scanning the patient over a certain time period to capture the dynamics of blood flow within the brain.^5^ From the resulting 4D perfusion scan, so‐called perfusion maps can be derived using dedicated software. These maps encode parameters such as cerebral blood volume (CBV), cerebral blood flow (CBF), or time to maximum enhancement (TMAX). They are used to assess the extent of stroke core and penumbra to decide about the treatment of the patient. But the automated assessment of core and penumbra based on these maps is prone to noise and artifacts, especially due to beam‐hardening at the base of the skull, and a manual delineation of these areas by radiologists is time consuming and subject to inter‐observer variability.^6^, ^7^ Despite these advances in medical imaging, many clinics lack access to advanced perfusion or multiphase imaging.^8^ Consequently, NCCT and single‐phase CTA remain the most widely implemented imaging techniques but are harder to interpret in terms of lesion extent. Technically, robust AI‐based segmentation tools that can operate reliably across different CT image types and sites could help bridge this gap, ensuring consistent assessment even when advanced scans or expert readers are unavailable.^9^ Accordingly, evaluating DL‐based segmentation performance across NCCT, CTA, mCTA, and CTP can help determine which imaging domain provides the most informative signal for stroke lesion segmentation. Because intravenous thrombolysis and endovascular thrombectomy are time‐critical and imaging‐driven, improved automated segmentation can support eligibility decisions or aid recognition on NCCT or CTA when perfusion imaging is unavailable, and reduce interobserver variability.^6^, ^10^ Beyond addressing this clinical gap, comparing NCCT, CTA, mCTA, and CTP is scientifically significant because these modalities rely on different contrast mechanisms and thus emphasize different aspects of the same pathophysiology. NCCT primarily shows parenchymal hypoattenuation and loss of gray–white matter differentiation, CTA depicts vessel occlusion, mCTA adds delayed phases that capture collateral filling, and CTP encodes perfusion dynamics.

Having outlined the clinical and technical motivation, the following paragraph introduces how deep learning and machine learning approaches have been applied to address these challenges, segmenting the affected regions on different CT images to possibly assist the clinical workflow. Segmenting stroke lesions on NCCT scans is possible as early ischemic changes due to increased water uptake can be visible. Several works have explored different DL‐based models to identify these changes automatically.^11^, ^12^, ^13^, ^14^, ^15^ Wang et al. utilized the SwinUNETR, featuring a self‐attention module as an encoder, a convolution‐based decoder, and additional uncertainty quantification modules^13^ to solve this problem, while Ni et al. introduce an asymmetry disentanglement network that generates a synthetic NCCT volume, compensating for intrinsic asymmetries and highlighting pathological ones, which is then used as input for a segmentation network.^15^ The model is trained and tested on the public AISD dataset that includes 345 training and 52 testing cases. Further works have investigated the use of CTA scans for stroke lesion segmentation.^16^, ^17^ Giancardo et al. also used a network taking into account symmetric considerations, called DeepSymNet‐v3. The two CTA brain hemispheres are separately forwarded into the network, which learns overall stroke core volume but also outputs a segmentation map using a strategy called weighted gradient‐based segmentation. They compared their method to the nnU‐Net^18^ with the CTA as a single input channel as well as a two channel input with the CTA and its left‐right flipped version. The CBF maps of the CTP generated by a commercial software were used for additional comparison. There hardly exists any research on the use of mCTA scans with DL models, although mCTA captures temporal collateral‐flow dynamics^19^ that may reflect tissue viability and could thus enhance lesion delineation. Wang et al. used NCCT, CTA, and a CTA+ scan (8 s after CTA) for stroke core segmentation with a 3D CNN using the scans as independent channels and comparing the output with CTP‐based segmentations.^20^ Using internal and external test cohorts of 345 and 108 patients, respectively, models trained with all three inputs outperformed those trained on a single imaging modality, suggesting the potential benefit of mCTA.

When using CTP scans for automated lesion segmentation with neural networks, different approaches exist using either the original perfusion series^21^, ^22^, ^23^, ^24^ or generated perfusion maps.^25^, ^26^, ^27^, ^28^, ^29^, ^30^ Soltanpour et al. use a MultiRes U‐Net to segment stroke lesions on perfusion maps, which is a modified version of the U‐Net.^28^ Different approaches were compared using only one of the CTP maps, a combination of maps as multiple channels or an added contra‐lateral version of the maps. While the use of CTP maps requires an additional preprocessing step to generate these maps and their outcome also varies between different vendors of the perfusion software, other works solely rely on the original CTP scan.^21^, ^22^, ^23^, ^24^, ^31^ De Vries et al. used CTP source images of the ISLES 2018 dataset to train and test their developed model, the PerfU‐Net.^24^ This network is a symmetry‐aware spatio‐temporal segmentation model that receives 2D+*t* data, which are hand‐picked slices of the CTP scan (with at least one lesion visible in the slice) at several time points. Investigating the length of the sequence, the authors demonstrated that reducing the temporal length from 32 to 16 time points did not affect the performance. While these models achieved comparable performance to others relying on CTP maps or NCCT scans, the best Dice score of 0.56 remains relatively low. Moreover, using individual slices of the perfusion scan omits useful spatial information. A concise overview of the prior works presented so far—including modality, dataset, and reported Dice performance—is provided in Table A.1.

**TABLE 1:** Dataset statistics for patients from Center A (91 cases) and B (166 cases).

	Sex		Age	Infarct volume				
	M	F		<1 mL	1–10 mL	10–70 mL	>70 mL	Mean ± std
Center A	42	49	71.2 ± 13.8	21	46	22	2	13.3 ± 18.3 ml
Center B	78	88	71.6 ± 13.6	25	43	77	21	34.5 ± 37.3 ml

*Note*: Cases are split into groups depending on the volume of the stroke core.

**TABLE 2:** Acquisition parameters for different CT image types from Centers A and B.

Center	Scan type	Tube voltage $\left[\text{kV}\right]$	Resolution $\left[{\text{mm}}^{3}\right]$	Slice width $\left[\text{mm}\right]$	# Slices
A	NCCT	90/150	0.45×0.45×1.0	1.5	163
	CTA	90/150	0.47×0.47×0.7	0.75	507
	mCTA	90/150	0.45×0.45×0.7	1.0	258
	Perfusion	70	0.39×0.39×3.0	5	37
B	NCCT	100 (150), 120 (14), 140 (2)	0.43×0.43×0.8	1.0	182
	CTA	80 (6), 100 (153), 120 (7)	0.63×0.63×0.5	0.5	673
	Perfusion	80	0.43×0.43×1.0	1.5	94

*Note*: Scans from Center B are acquired with a Dual Energy Scanner. Median values are given for the resolution, slice thickness, and number of slices. The image size is 512×512 across all scans. The number of patients for each tube voltage level is given in parentheses.

**TABLE 3:** Results of data harmonization.

	CT scan type	Resolution $\left[{\text{mm}}^{3}\right]$	Slice thickness $\left[\text{mm}\right]$	# Slices
Center A	NCCT	0.39×0.39×1.0	1.5	111
	CTA		0.75	
	mCTA		5	
	Perfusion		5	
Center B	NCCT	0.43×0.43×1.0	1.0	94
	CTA		0.5	
	Perfusion		5	

*Note*: Median values are given for the resolution. The image size is 512×512 across all scans.

**TABLE 4:** Modality comparison: Training and testing on different CT image types from Center A to segment stroke core.

Training and test data Center A	Volume Group 1<x<10	Volume Group 10<x<70	p<0.05
NCCT	0.16±0.23	0.36±0.28	CTA, mCTA, CTP
CTA	0.21±0.27 (+31.3%)	0.55±0.27 (+52.8%)	NCCT, mCTA, CTP
mCTA	0.33±0.31 (+106.3%)	0.71±0.22 (+97.2%)	NCCT, CTA
CTP	0.32±0.30 (+100.0%)	0.78±0.09 (+116.7%)	NCCT, CTA

*Note*: Mean (±std) modified Dice values for different infarct sizes and difference compared to NCCT in percentage. The leading Dice values are marked in bold. CTP time points are selected as in an mCTA protocol. Wilcoxon signed‐rank test with Bonferroni correction was performed to assess statistically significant difference between the models (*p<0.05). Detailed *p*‐values in Table A.2.

**TABLE 5:** Site‐transfer results: modified Dice values (mean ± std) for segmentation of stroke core with the best values marked in bold.

Model training Center B	Fine‐tuning Center A	Testing Center A	Volume Group 1<x<10	Volume Group 10<x<70	*p*‐value
NCCT		NCCT	0.14±0.20	0.42±0.29	0.00000*
NCCT	✓	NCCT	0.22±0.21	0.57±0.22	
CTA		CTA	0.19±0.24	0.60±0.21	0.00007*
CTA	✓	CTA	0.31±0.26	0.67±0.17	
CTP		mCTA	0.37±0.30	0.72±0.18	0.01256*
CTP	✓	mCTA	0.40±0.28	0.77±0.12	
CTP		CTP	0.46±0.28	0.76±0.09	0.00239*
CTP	✓	CTP	0.50±0.27	0.80±0.10	

*Note*: Direct inference of Center A data on the Center B‐trained model versus additional fine‐tuning on Center A data. The *p*‐values are calculated using the Wilcoxon signed‐rank test.

* p<0.05.

**TABLE 6:** Investigating the influence of time point selection of perfusion time points: modified Dice values (mean ± std) for segmentation of stroke core for mCTA and different CTP timing scenarios. Bold indicates the best modified Dice value per volume group.

Data for Training/testing		Volume Group 1<x<10	Volume Group 10<x<70
*mCTA*	Number of channels – timing	0.33±0.31	0.71±0.22
Perfusion	3 – “Peak” timing	0.38±0.29	0.72±0.13
Perfusion	3 – Fixed mCTA timing	0.32±0.30	0.78±0.09
Perfusion	3 – Varying mCTA timing	0.27±0.30	0.75±0.13
Perfusion	4 – “Peak” timing + native baseline	0.50±0.27	0.73±0.14
Perfusion	8 – Equidistant	0.46±0.32	0.73±0.21

The most prominent and crucial concerns regarding such DL‐based methods revolve around their generalizability to unseen data as models tend to overfit on specific characteristics of the training dataset.^32^ There exist considerable differences in the data from various clinical sites, due to different scanners, imaging protocols and acquisition parameters, patient cohorts, prevalence, and extent of, for example, stroke lesions. These variations can lead to spurious correlations and biases, challenging the robustness of DL models in diverse clinical settings. Recent work has addressed the challenge of domain transfer between clinical sites, particularly on magnetic resonance (MR) images, through approaches like the self‐adaptive normalization network (SAN‐Net).^33^ This network, featuring masked adaptive instance normalization and symmetry‐inspired data augmentation, has demonstrated superior generalization across multiple sites, outperforming existing methods on the ATLAS dataset.

As summarized in Table A.1, existing DL‐based approaches predominantly focus on a single CT image type or limited combinations, are typically trained and evaluated on single‐site datasets, and report only moderate Dice scores. Therefore, the main contribution of our work lies in the comprehensive comparative evaluation of different CT image types involved in stroke imaging—NCCT, CTA, mCTA, and CTP—for DL‐based stroke lesion segmentation. In addition, we investigate the model transferability across clinical sites, addressing the challenges posed by varying imaging protocols and patient cohorts. A further novel aspect is the analysis of CTP timing strategies, investigating how different selections of time points from the 4D CTP series affect segmentation performance. Specifically, this study addresses three core research questions: (i) How does DL‐based stroke segmentation performance compare across the main CT image types (NCCT, CTA, mCTA, and CTP) within a unified framework? (ii) How well do models trained on one clinical site generalize to unseen sites with differing scanners and protocols, and can limited fine‐tuning improve cross‐site performance? (iii) How does the selection of distinct time points from 4D CTP data influence model performance?

To address these questions, we employ nnU‐Net as a strong and widely used medical image segmentation network with automated configuration, enabling a robust and reproducible baseline for comparisons across CT image types.^11^, ^34^, ^35^ As we have data available from two different clinical sites, stemming from two different continents, we aim to quantify the model performance in terms of generalizability. To this end, we perform regular inference of data from an unseen clinical site on an existing model and try to improve the performance by fine‐tuning the model on a subset of the unseen data. To address the challenge of processing the initially large series of images and being independent of vendor‐specific perfusion map software, we study the impact of time point selection from the 4D CTP data with the use of nnU‐Net. Overall, this study aims to provide a unified comparison of CT image types and to offer practical insights into the generalization and robustness of DL‐based stroke segmentation that can inform clinical decision‐making.

The remainder of this paper is organized as follows: Section 2 describes the datasets and methods, Section 3 presents quantitative and qualitative results, Section 4 discusses implications and limitations, and Section 5 concludes the study.

## 2 MATERIALS AND METHODS

### 2.1 Dataset

The scans used in this work were collected from multiple retrospective studies which each received Institutional Review Board approval prior to starting. The requirement for informed consent was either obtained or waived. The main dataset comprises 91 stroke patients examined at the Vancouver General Hospital (Center A), Vancouver, Canada, with a Somatom Force Scanner (Siemens Healthineers, Forchheim, Germany) including NCCT, (multiphase) CTA, and CTP scans. Patient information as well as stroke lesion volumes are listed in Table 1. Scanning parameters, that is, tube voltage, image resolution, and slice thickness can be found in Table 2. The image dimensions are 512×512 pixels for all scans. The mCTA protocol consists of an initial scan that is regarded equivalent to a conventional arterial CTA scan. Following the first acquisition, there is a late arterial and late venous phase scan acquired, 13 and 21 s after the initial CTA scan, respectively. For most patients (85 cases), the perfusion scan consisted of 44 images acquired at 1.5‐s intervals, resulting in a total scan time of 66 s. For six cases, only 25 images were acquired, corresponding to a scan duration of 38 s. From the perfusion scans, perfusion maps encoding CBV, CBF, and TMAX were calculated and used for annotation support. From the 91 patients, 13 were identified with only penumbra and without any signs of stroke core, while another six patients showed signs of prior infarction but no acute stroke.

NCCT, CTA, and perfusion scans from a different site, Universitätsklinikum Schleswig‐Holstein (Center B), Lübeck, Germany, were used in parts of the experiments to assess domain transfer capabilities in stroke segmentation. The scans from this dataset were acquired using a Somatom Definition AS+ scanner (Siemens Healthineers, Forchheim, Germany) and include 166 patients. In contrast to the data from Center A, only conventional (single‐phase) CTA scans were performed at Center B and the perfusion protocol involves a scan time of 45 s (30 time points). Patient information and acquisition parameters can be found in Tables 1 and 2. Within this cohort, stroke core could not be identified in 24 patients, while nine patients also showed no evidence of penumbra in the perfusion maps.

### 2.2 Labeling scheme

**FIGURE 1:**
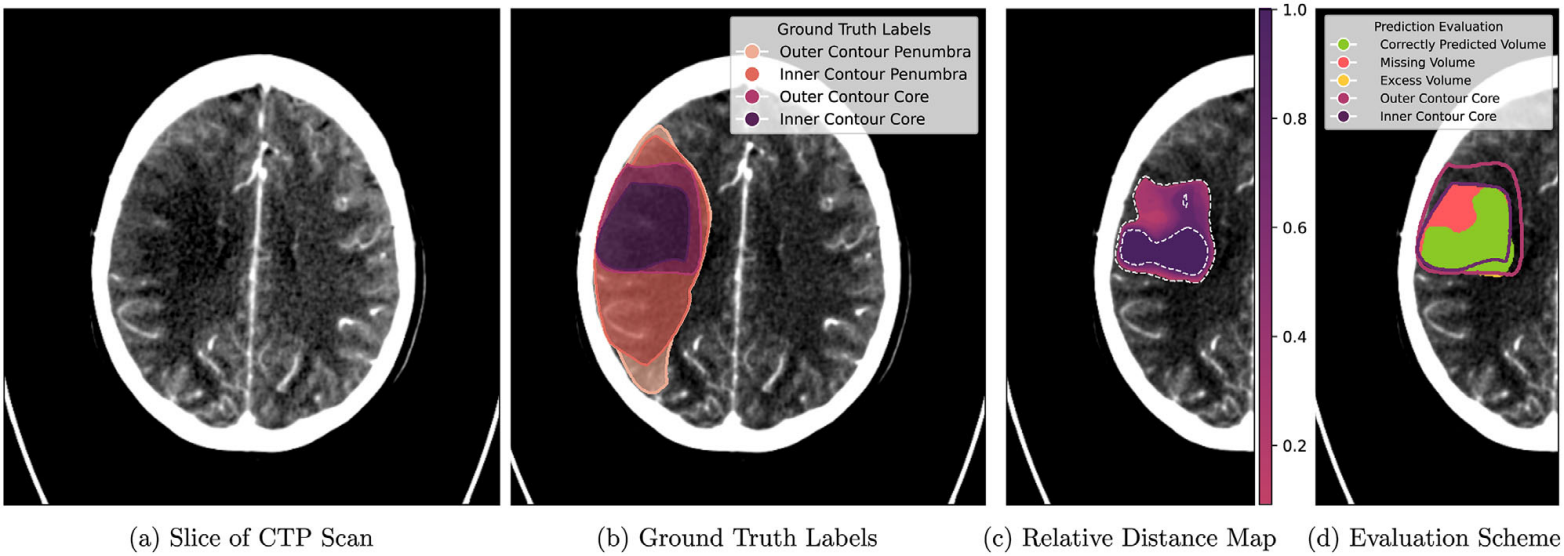
(a) Slice of the original CTP image. (b) Ground truth of core and hypoperfused tissue (union of core and penumbra) with inner and outer boundary overlay on CTP. (c) Relative distance map of the post‐processed prediction for stroke core. The relative distance map is derived from the Euclidean distance transform of the predicted inner and outer boundaries, normalized to [0,1]. Voxels near the predicted inner contour have values approaching 1, whereas voxels near the predicted outer boundary approach 0. (d) Prediction shown in (c) thresholded at 0.5 and compared to ground truth labels of infarct core (same contours as in (b)). Correctly predicted region in green, missing region in red, and excess volume in yellow.

The labels used to train segmentation models in this work account for the uncertainty inherent to the task of stroke segmentation as described in Vorberg et al.^36^ Based on the perfusion maps derived from the original perfusion scan—CBV, CBF, and TMAX—inner and outer contours delineate the minimum and maximum extent of core and hypoperfused volume. Hypoperfused volume denotes the entire perfusion deficit (i.e., the union of core and penumbra). Using this scheme, the resulting label constitutes a nested inner and outer contour for both core and hypoperfused volume (see Figure 1b) with the tube‐like structure between the boundaries denoting an uncertain region. First, a preliminary core and penumbra evaluation is performed automatically with the syngo.via CT Neuro Perfusion application based on thresholds applied to the perfusion maps. These initial boundaries are then further refined manually, taking into account CBV, CBF, and TMAX as well as typical artifacts, for example, at the base of the skull, and ensuring anatomical plausibility and compactness. Figure 1a shows an example of a CTP scan and a version overlayed with its labels according to the described scheme in Figure 1b. Additionally, prior infarcts are delineated with a single contour to support the training process as an auxiliary task. Providing this class label, the network learns to distinguish old infarcts from acute ones. This label is handled as its own segmentation target and is not part of the hierarchical inner/outer structure used for the acute core and hypoperfused volume. At the voxel level, all classes (acute core/hypoperfusion, and prior infarct) are encoded as mutually exclusive in accordance with nnU‐Net's labeling scheme.

The segmentation labels are directly created on the baseline time point (2nd time point) of the motion correction algorithm of the perfusion series, while the other scans, NCCT, CTA, and mCTA, are aligned with this volume using a rigid registration algorithm. For the perfusion series itself, the 3D volumes of all time points are registered to the baseline time point as well using rigid registration.

### 2.3 Experimental setup

#### 2.3.1 Data harmonization

To harmonize the datasets and enable the transfer of annotations derived from the perfusion images, several preprocessing steps were applied. For the data from Center A, CTP had a relatively low resolution in *z*‐direction, compared to the other modalities. To preserve the higher resolution of the NCCT, CTA, and mCTA scans, the CTP data were upsampled to a slice spacing of 1 mm. Subsequently, NCCT, CTA, and mCTA images were registered and resampled to the new perfusion reference grid with a median resolution of $0.39\times 0.39\times 1.0\;{\text{mm}}^{3}$ (see Table 3). The voxel dimensions varied slightly in the *x*–*y* plane while the *z* dimension was constant across all cases of the dataset. The registration and resampling steps are implemented in our publicly available repository.^37^ To enable direct comparison between CTP and mCTA scans, despite their differing slice thicknesses of 5 and 1 mm, respectively, we synthesized thicker mCTA slices. Therefore, several thin mCTA slices, with an initial slice thickness of 1 mm, were averaged before resampling the mCTA volume to the reference grid resulting in 5‐mm slice thickness. The resulting input image dimensions across all modalities were 512×512×111.

For Dataset B, the CTP images were acquired with a slice spacing of 1 mm, and therefore no upsampling was required. The corresponding NCCT and CTA scans were registered and resampled to the CTP reference grid without further adjustment. To maintain consistency with Dataset A, thick slices of 5 mm were synthesized on the CTP scans in Dataset B. For the remaining modalities, the slice thickness was comparable between datasets (Table 2), so no additional adaptations were necessary and the image size for all modalities in Center B was 512×512×94. The exact details of the data after preprocessing are listed in Table 3.

#### 2.3.2 nnU‐Net for stroke segmentation

All conducted experiments are based on nnU‐Net^18^ as a state‐of‐the‐art medical segmentation network. It has proven superior compared to other architectures, as, for example, Soliman et al.^38^ analyzed in their work comparing stroke segmentation performance between nnU‐Net and other segmentation networks such as the transformer‐based DAE‐Former and a CNN‐based Large Kernel Attention network in MRI‐based stroke lesion segmentation. In the CT stroke segmentation context, nnU‐Net has likewise been adopted as an established method in multiple studies, including NCCT‐based infarct segmentation.^11^, ^34^, ^35^ Furthermore, a recent comprehensive benchmark^39^ demonstrates that well‐configured U‐Net models still outperform many transformer architectures when rigorously evaluated. Given the moderate size of our datasets and our focus on modality‐ and site‐comparisons, nnU‐Net offers a robust and reproducible framework with minimal additional tuning. Therefore, we use nnU‐Net to obtain a reliable estimate of the potential of a DL‐based system for stroke segmentation and compare its performance across different CT imaging types.

nnU‐Net is a self‐configuring framework that first extracts a fingerprint from the data used for network training, which then determines a set of rule‐based parameters concerning data preprocessing and network configuration. The images are normalized using CT normalization, which means that the image values are clipped to the [0.5, 99.5] percentiles of the intensity values covered by the segmentation labels, followed by *z*‐score normalization based on mean and standard deviation of all intensity values in the image. The optimizer is stochastic gradient descent (SGD)^40^ with Nesterov momentum and the learning rate is a poly learning rate schedule with an initial value of 0.01. The loss function is a combination of soft dice loss and cross‐entropy loss.^41^ The model is trained for 1000 epochs, following the standard nnU‐Net setting, with a batch size of 2 in a five‐fold cross‐validation scheme to predict both inner and outer contours of stroke core and hypoperfused area from different CT images as input. Additionally, prior infarctions were segmented as an auxiliary task as part of a multiclass problem. As the labels follow a hierarchical order, that is, core is surrounded by penumbra and the inner boundary lies within the outer boundary, the region‐based training scheme of nnU‐Net was employed. Instead of mutually exclusive classes, binary region masks derived from merged labels are learned. Concretely, voxels inside the inner contour are positive for both the inner‐ and outer‐core region masks, whereas voxels in the annular “tube” are positive only for the outer mask. The hierarchy is encoded as region targets, and the standard Dice + cross‐entropy loss is applied per region channel. In this way, regions of merged labels are learned rather than individual labels, while the prior infarct label is handled as a separate, nonhierarchical class.

No custom modifications were made to nnU‐Net, and multiphase inputs were provided simply as multiple channels, which nnU‐Net handles in the same way as standard multimodality inputs. All experiments were run on a single NVIDIA Tesla V100 GPU (32GB VRAM) under PyTorch 2.0.1 with CUDA 11.7. Training used nnU‐Net v2 in 3D full‐resolution mode for 1000 epochs and mean epoch time varied between modalities (≈130–266 s/epoch), primarily due to differing CPU availability for data loading on the shared cluster.

#### 2.3.3 Modality comparison

**FIGURE 2:**
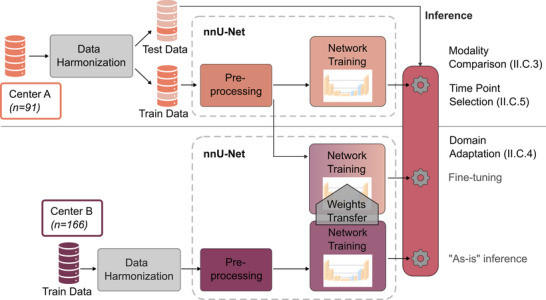
Flowchart of the experimental pipeline used for every modality. Data harmonization comprises registration to the CTP reference, slice‐thickness adaptation, and reformatting to a common image size as described in Section 2.3.1, whereas subsequent preprocessing is performed within the nnU‐Net framework (Section 2.3.2) based on the dataset “fingerprint.” The upper part shows the Modality Comparison experiments on data from Center A (Section 2.3.3); the same scheme is used for the Time Point Selection experiments (Section 2.3.5). The lower part illustrates the Domain Adaptation setup (Section 2.3.4), where a model pretrained on data from Center B is either applied as‐is to Center A or further fine‐tuned on Center A data with weight transfer. The upper branch of the pipeline is illustrated for one fold of the five‐fold cross‐validation, whereas pretraining on Center B is performed once using the full dataset.

The first part of the experiments assesses the ability of a DL‐based segmentation model to leverage information for stroke lesion segmentation available in different types of CT images. Therefore, nnU‐Net is trained using a single imaging modality at a time—NCCT, CTA, mCTA, or CTP images—and tested on images from the same type. The upper part of Figure 2 depicts the experiment pipeline, which is identical for every modality and is shown here for one of the five cross‐validation folds. NCCT and CTA datasets, comprising single‐phase acquisitions, were input as one channel into the network. In contrast, mCTA, which includes three phases, was input concatenating the images in channel dimension. To align mCTA with CTP for comparison, we selected three perfusion phases analogous to the mCTA timing. From the motion‐corrected perfusion series, time‐attenuation curves were calculated on an ROI of an arterial and reference vessel. Hereby, the arterial input function (AIF) and a venous reference vessel (superior sagittal sinus) are automatically determined by the syngo.via CT Neuro Perfusion software (Siemens Healthineers). Mimicking mCTA's initial bolus‐triggered phase, we chose the second‐last time point before the peak arterial enhancement from the perfusion data, averaging it with the two surrounding scans to define the initial perfusion phase. Subsequent late arterial and venous phases were similarly derived and averaged, maintaining consistent intervals. These three phases were concatenated to form the input channels for nnU‐Net training.

#### 2.3.4 Domain adaptation experiments

The second part of the experiments explores domain adaptability, an important aspect for the applicability of DL models in clinical practice. To assess how well a model trained to segment infarcts on data from one clinical site is able to perform the task on data from an unseen site, data from Center A is directly input into nnU‐Net trained with data from Center B, as illustrated by the lower branch in Figure 2. One approach to possibly improve the performance is an additional fine‐tuning of the existing model on a subset of the new data (middle branch of the pipeline in Figure 2). In this experiment, the initial model trained with the standard nnU‐Net setting on data from Center B is used, keeping its final weights. The fine‐tuning process is started on the dataset from Center A, leaving out 20% of the data for testing. During fine‐tuning, the network is only trained for 200 epochs, as convergence was reached within this range, instead of 1000 and the initial learning rate is set to the value of the final learning rate at the end of the initial network training. Fine‐tuning is again conducted in a five‐fold cross‐validation scheme, always retaining 20% of the data for testing to obtain averaged evaluation metrics over the whole dataset of Center A. The performance of this approach is then compared to the first scenario.

For both approaches—as‐is inference and fine‐tuning—all four CT image types are used again. As Center B does not include mCTA data, the model used for inference (fine‐tuning) of the mCTA data from Center A is trained with CTP data. By using the proposed time point selection scheme and also synthesizing thick slices on the perfusion scans from Center B, as described in Section 2.3.1, this setup is assumed to resemble the mCTA data best.

#### 2.3.5 Time point selection experiments

**FIGURE 3:**
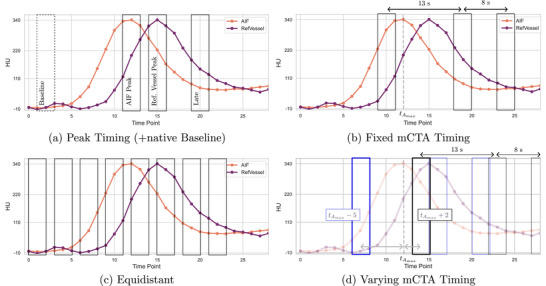
Timing scenarios: HU values in an arterial vessel (AIF) and a reference vessel are plotted across time points. Rectangles present the averaging of three subsequent scans. (a) Peak timing with additional native baseline. (b) Fixed mCTA timing with 13 and 21 s delay after the initial phase (like in the mCTA protocol). (c) Equidistant timing—using eight subsequent nonoverlapping windows of three scans. (d) Varying mCTA timing: mCTA‐like timing with a random sampling of the first time point around the AIF maximum ($t_{A_{max}}$).

**FIGURE 4:**
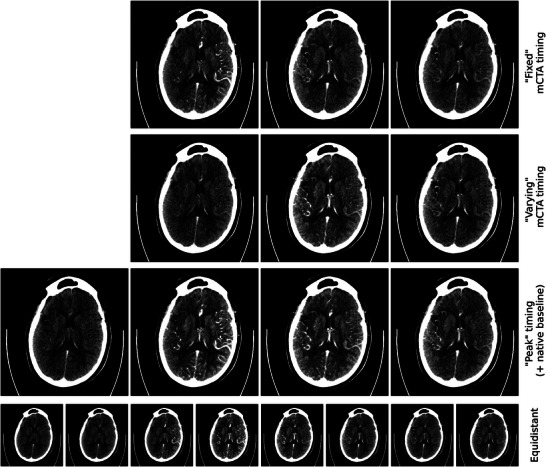
Visual example of time point selection. Each phase consists of three averaged consecutive scans. Windowing is C 75 HU/W 100 HU for all images. Top row: “Fixed” mCTA timing. Second row: “Varying” mCTA timing (the offset around the arterial peak time point differs between patients). Third row: “Peak” timing with an additional native baseline phase. Bottom row: “Equidistant” timing with eight nonoverlapping windows of three scans each.

To investigate the potential of perfusion images for stroke segmentation even further, the last experiment block focuses on the time point selection for the CTP phases fed to the network. While the timing of the phases is fixed for the mCTA scans, different scenarios can be created using the time‐attenuation curves of the arterial input and a reference vessel to determine the ideal timing. A visualization of all timing scenarios is depicted in Figure 3. In general, three consecutive scans, centered around specified time points, were averaged to obtain one perfusion phase. For clarity and consistency throughout this process, we refer to the central time point of these three images as the “reference time point.” For the mCTA‐like time point selection, the initial phase is set to the second last time point before the arterial peak enhancement. As described in Section 2.3.3, the same time intervals between the phases as in the mCTA protocol were used, 13 and 21 s after the initial phase, which is denoted as “fixed mCTA timing” (Figure 3b and top row of Figure 4).

In a second scenario, both time‐attenuation curves were utilized. The first reference time point is set to the point of maximum enhancement in the arterial input and the second window is centered around the point of maximum enhancement in the reference vessel. The reference time point for the last phase is then determined by adding the time interval between the first and second reference time point to the second phase reference time point. This experiment is referred to as “peak timing.” Extending this approach, an experiment with four input channels (“peak” timing + native baseline) is conducted using an additional “baseline non‐contrast phase” by choosing the third time point of the perfusion series as reference time point. Both scenarios are shown in Figure 3a and in the third row of Figure 4.

A third scenario with three phases also mimics the mCTA scenario, but in a more realistic way. As the actual arterial peak is unknown in the acquisition of an mCTA, a small random timing offset around the true arterial peak $t_{A_{\max}}$ is introduced. Specifically, the reference time point of the first phase is set to $t_{0}=t_{A_{\max}}+\delta$, with a random offset δ chosen uniformly from −5 to +2 time points ($t_{0}\in \left[t_{A_{\max}}-5;t_{A_{\max}}+2\right]$). The second and third phases then follow the mCTA protocol at $t_{0}+13$ s and $t_{0}+21$ s, respectively (Figure 3d). This construction reflects interpatient variability in bolus arrival and heart rate while keeping the interphase spacing consistent with mCTA practice, which is denoted as “varying” mCTA timing in the following. A visual example of this timing strategy is shown in the second row of Figure 4, in contrast to the “fixed” mCTA timing in the top row, where the first phase is fixed to two time points before arterial peak enhancement.

For all three timing setups described, if the initially calculated window for the last phase would lie outside the range of available scans, its reference time point is set to the second last time point of the series. To investigate the benefit of using more time points, the whole perfusion series is used, concatenated in channel dimension. For the first 24 time points, groups of three consecutive time points each were averaged ($t\in [t_{0};t_{23}]$) resulting in eight phases to be used as network input (Figure 3c and exemplary case in bottom row of Figure 4). This experiment is referred to as “equidistant” in the following. Some CTP scans comprise more time points, but as the number of channels has to be fixed, the minimum of time points (25) had to be taken, dividing it by three to obtain the maximum number of channels. The overall training and evaluation setup for the time point selection closely follows the modality comparison experiments and is illustrated in the upper branch of Figure 2.

### 2.4 Evaluation

To evaluate the network predictions, we generate iso‐contours for both core and hypoperfused volumes based on the relative distance between the predicted inner and outer boundaries. The Euclidean distance transform (EDT) is used to compute the distance of each voxel to the nearest boundary, which is then normalized to obtain a relative distance map $d_{r}\in \left[0,1\right]$, where values close to 1 lie near the predicted inner contour and values near 0 lie close to the predicted outer contour. Thresholding this map yields a family of iso‐contours. For each threshold, the predicted contour is compared to the ground‐truth region, following the uncertainty‐aware scheme in Vorberg et al.^36^ We compute correct, missing, and excess volume with respect to this region. A modified Dice score, accounting for boundary uncertainty, is computed for each region, with results reported for the core region using the best threshold based on average performance across cases. The modified Dice score proposed in Vorberg et al.^36^ treats voxels lying inside the ground‐truth outer boundary as nonpenalized excess, thereby accounting for annotation uncertainty. The modified Dice for a prediction A and inner/outer ground‐truth contours $B_{I},B_{O}$ is as follows:

(1) $$D\left(A,B_{I},B_{O}\right)=\frac{2\cdot \left|A\cap B_{O}\right|}{\left|A\right|+\left|B_{I}\right|+\left|A\cap (B_{O}\setminus B_{I})\right|}.$$

To assess boundary accuracy, we additionally compute the average symmetric surface distance (ASSD).^42^ For predicted surface $S_{A}$ (using the same threshold on the EDT as for the modified Dice score) and ground‐truth surface $S_{B}$ (the union of inner and outer annotated contours), ASSD is defined as follows:

(2) $$\begin{array}{cc} \mathrm{ASSD}(S_{A},S_{B}) & =\frac{1}{|S_{A}\left|+\right|S_{B}|}\left(\sum_{a\in S_{A}}d\left(a,S_{B}\right)\right.\\ & \;\left.+\sum_{b\in S_{B}}d\left(b,S_{A}\right)\right), \end{array}$$

where d(x,S) is the shortest Euclidean distance from point x to surface S. To compare the performance of the models, we computed the pairwise Wilcoxon signed‐rank test and, when comparing more than two models, applied Bonferroni correction for multiple comparisons.

## 3 RESULTS

### 3.1 Modality comparison

**FIGURE 5:**
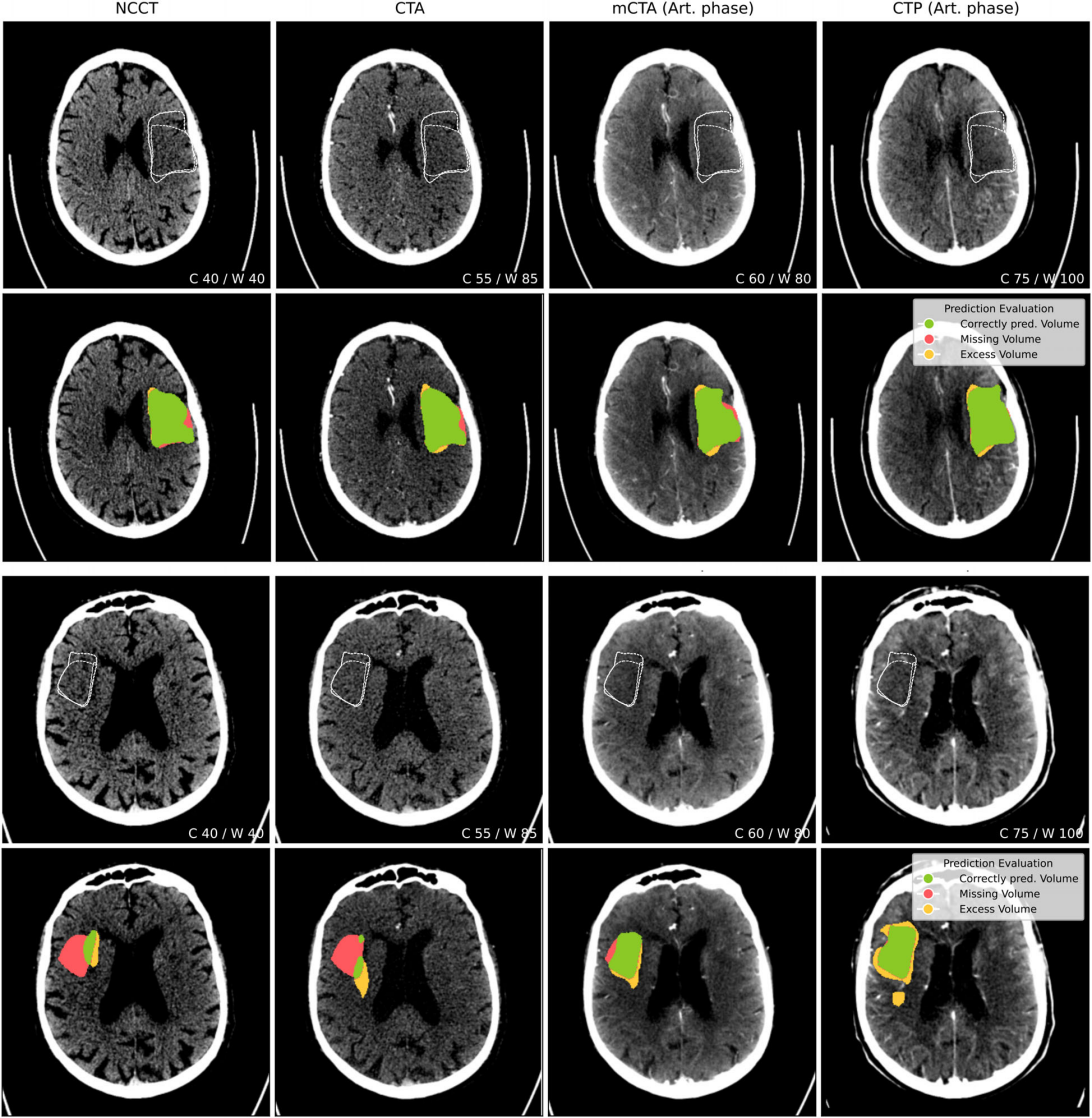
Two example cases (Center A)—modality comparison: Ground truth inner and outer boundary for stroke core are delineated in the upper row. Evaluation of the prediction as overlay in the lower row. The window settings for each image type are given in the lower right corner.

**FIGURE 6:**
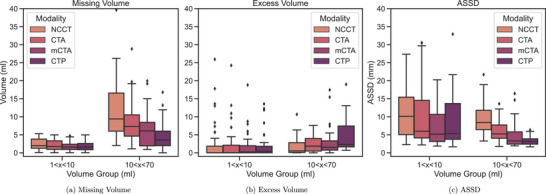
Missing and excess volume and average symmetric surface distance (ASSD) for stroke core predictions compared with GT annotations for the most frequent volume groups across all CT scan types.

The first part of the experiments investigates the performance across different CT image types used for training and testing nnU‐Net to segment stroke core on the data from Center A. An exemplary case including all modalities and the corresponding model predictions is presented in Figure 5. Table 4 shows the modified Dice values for the volume groups 1–10 and 10–70 mL, together with the difference (in percentage) of the modified Dice compared to NCCT. Following Ostmeier et al.,^7^ infarcts with a segmented volume below 1 mL were excluded from the analysis, as such very small volumes are unlikely to be reliably delineated in CT scans and have been considered of limited practical relevance in previous segmentation studies. This leads to 46, 22, and 2 cases remaining in the respective subgroups (1–10, 10–70, and >70 mL). As it is not meaningful to assess and report mean and standard deviation of two cases, the results for the >70‐mL subgroup are omitted in the following. Nevertheless, large infarcts remain an interesting subgroup, yet our study population does not provide enough samples for a proper evaluation. The overall best modified Dice score of 0.78 is achieved for the 10–70‐mL subgroup using CTP scans. For smaller infarcts of 1–10‐mL size, using mCTA data reaches a modified Dice of 0.33. Training and testing the model with NCCT scans exhibit the lowest dice scores across all volume groups. Considering the volumetric assessment of the predicted segmentations shown in Figures 6a and 6b, it can be observed that the missing volume is always larger than the excess predicted volume across all experiments, meaning that the infarct core is rather underestimated than overestimated. Figure 6c shows the ASSD between the prediction and ground truth infarct per volume group and modality. The ASSD is generally larger and the distribution more wide‐spread for the 1–10‐mL group. Comparing the different modalities, especially in the 10–70‐mL subgroup, it is visible that the prediction improves with respect to missing volume and ASSD when moving from NCCT to CTA, mCTA, and CTP, although the Dice score does not show a statistically significant improvement comparing mCTA and CTP. This also becomes apparent in the case example in the lower half of Figure 5, where the infarct is severely underestimated using the NCCT scan whereas the predicted segmentation is closely aligned with the ground truth on the CTP scan. When averaging over all infarcts ≥1 mL, the overall modified Dice shows the same trend, with NCCT 0.23±0.26, CTA 0.33±0.31, mCTA 0.45±0.33, and CTP 0.47±0.32.

### 3.2 Domain transfer results

**FIGURE 7:**
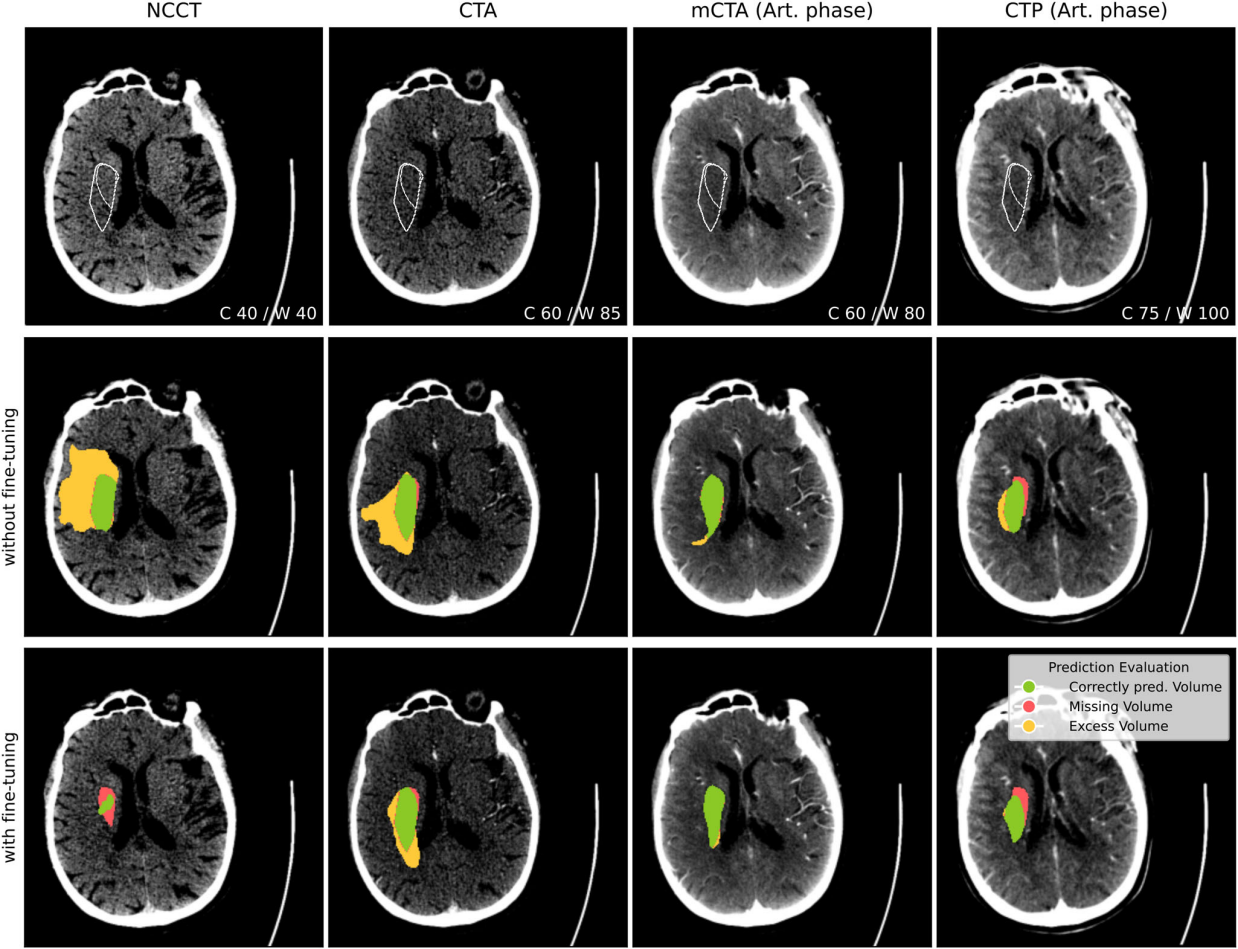
Example case, with and without fine‐tuning on Center A: Ground truth inner and outer boundary for stroke core are delineated in the upper row. Evaluation of the prediction as overlay in the second (without fine‐tuning) and third row (with fine‐tuning). The window settings for each image type are given in the lower right corner.

An exemplary case illustrating the effect of cross‐site transfer and fine‐tuning for all modalities is shown in Figure 7. In Table 5, the quantitative results for the experiments are shown. For comparison, scans from Center A are either directly inferred using the model trained on Center B or the pretrained model from Center B is fine‐tuned on data from Center A before inference. The modified Dice values are presented for each image modality and across the subgroups, together with the *p*‐value assessed with the Wilcoxon signed‐rank test. Across all the modalities and subgroups, the additional fine‐tuning improved the performance on the segmentation of stroke core significantly. The qualitative example for this analysis (Figure 7) shows that the infarct is over‐estimated for NCCT and CTA scans when directly performing inference on the model trained on Center B, while the prediction is smaller and closer aligned with the ground truth when fine‐tuning the model on data from Center A first.

### 3.3 Time point selection

Regarding CTP, several experiments were conducted involving different strategies for the selection of time points from the scan. Table 6 presents the modified Dice scores for the different timing scenarios for both remaining subgroups. The results for the mCTA experiment are included in the first row, as this experiment also involves training with multiple channels. For small infarcts (1–10  mL), using four time points—a base time point, arterial peak, reference vessel peak, and a late time point—achieves the highest performance with 0.5. The highest modified Dice of 0.78 is reached within the 10–70‐mL volume group for the experiment with a fixed timing as in an mCTA protocol. In comparison, training with the original mCTA, which has a nearly equal timing, only yields a modified Dice of 0.71 for the same subgroup. Considering all infarcts in the two remaining volume groups, the experiment using four time points exhibits a statistically significant difference in performance (p<0.05 in the Bonferroni‐corrected Wilcoxon signed‐rank test) compared to the mCTA (p=0.038), varying mCTA timing (p=0.017) and “peak” timing (p=0.001), while the difference in the Dice of the mCTA is only statistically significant compared to the four time point (p=0.038) and equidistant timing (p=0.011) but not the other scenarios.

## 4 DISCUSSION

In this work, we explore the use of different CT image types—NCCT, CTA, mCTA, and CTP—for stroke lesion segmentation using nnU‐Net. To facilitate a direct comparison of performance across these modalities, we utilize a dataset comprising all scan types from 91 patients, all examined at a single site. The predicted stroke core (and hypoperfused area) is compared to manual reference labels, which were annotated using the CTP maps and incorporate segmentation uncertainty. Based on this evaluation, modified Dice scores and volumetric measurements are reported. To further assess the transferability between clinical sites—an essential aspect for DL‐based solutions in medical imaging—we employ an additional dataset from a second site consisting of 166 cases. To this end, inference of data from an “unseen” site is either performed directly using a fully trained model or a model fine‐tuned on the new site before testing. The last part of the experiments focuses on the benefit of CTP data for stroke core segmentation using the original perfusion scan series. To reduce the size of the data for the use in a neural network, different time point selection scenarios are proposed and compared.

In the comparison of segmentation performance between the different CT image types, the general trends align with known imaging characteristics and our expectations regarding research question (i): CTP and mCTA yield the best modified Dice scores (Table 4) and outperform NCCT and CTA. The reason for lower segmentation performance on NCCT might be the subtlety of parenchymal changes in the early hours of onset which makes infarcts harder to detect. CTA adds vascular information but still lacks temporal perfusion contrast. mCTA and CTP, in contrast, reflect delayed collateral filling and perfusion dynamics, respectively, which produce clearer lesion‐related signal changes and therefore support more accurate delineation. These observed trends are in line with the work of Wang et al.^20^ showing improved segmentation performance going from NCCT to CTA to CTA+, which is a late‐phase CTA (8 s post‐initial scan), based on scans derived from a perfusion series. In terms of absolute performance, direct comparison is limited by differences in datasets and by our uncertainty‐aware metric. But, the observed accuracy is broadly consistent with prior studies, for example, Wang et al.^13^ report Dice values in a similar range for NCCT‐based stroke segmentation (0.31–0.37 for infarcts ≤40 mL).

Regarding the volumetric assessment in Figure 6, the missing volume is nearly always larger than the excess predicted volume, with the largest underestimation observed for NCCT, followed by CTA. This trend is also visible in the case example in the lower half of Figure 5, where the infarct is barely visible on NCCT and thus severely underestimated, while predictions on mCTA and CTP are much closer to the ground truth. This likely reflects cases imaged early after stroke onset, when hypoattenuation has not yet fully developed, making the infarct difficult to distinguish from normal tissue. In line with this, the model tends to behave conservatively: In regions where no image features clearly indicate pathology, it defaults to predicting healthy tissue, which constitutes the majority of the brain volume. In contrast, in the example in the upper half of Figure 5, more time has likely passed since stroke onset. Here, the ischemic change is more readily visible in the NCCT and could therefore also be detected more accurately, resulting in a similar prediction compared to the more advanced scans, like mCTA and CTP.

To investigate the transferability of stroke segmentation between clinical sites, we performed direct inference on nnU‐Net trained on data from Center B and compared it to a fine‐tuning approach where the model is additionally trained for a smaller number of epochs on data from Center A before testing. For small‐ and middle‐sized infarcts, the fine‐tuned model outperformed the standard model across all modalities, supporting our expectation in research question (ii) that cross‐site generalization improves with site‐specific fine‐tuning. Generally, the potential for improvement of the performance is larger for more difficult tasks such as the segmentation based on NCCT scans, whereas the infarct is easier to identify on CTP scans. Here, a fine‐tuned model can only boost the performance to a certain extent, which is also visible in Figure 7, where the correctly predicted area does not differ much between the experiments except for NCCT.

As an aside, the nnU‐Net framework suggests a slightly different pretraining and fine‐tuning approach where the target dataset has to be known before the initial training phase. In the experiment planning stage of nnU‐Net, hyperparameters such as patch size and network topology are optimized for the dataset. For optimal fine‐tuning results, experiment planning is done for the target domain first and the resulting plan is used for training on the source domain. Experiments involving pretraining and finetuning including moving the experiment plans between the datasets were also conducted but delivered worse results. In addition, the scenario used in our work, which follows a standard transfer‐learning strategy, is flexible with respect to the target domain as the same base model can be independently fine‐tuned with various target datasets.

Exploring the use of CTP data for stroke core segmentation showed that the selection of the phases from the perfusion series has an influence on the performance of the model. For middle‐sized infarcts, the difference in the modified Dice score is small with the best value of 0.78 for the fixed mCTA timing scenario. Although this time point selection was designed to resemble an mCTA acquisition, there is still a performance gap in the modified Dice score with CTP outperforming the mCTA (0.78 vs. 0.71). The results hint at a possible influence of the start timing of the mCTA that is usually bolus‐triggered but depends on the cardiac output of the patient. The performance of the fixed mCTA timing experiment shows that a well‐aligned mCTA timing works well, but the performance drops as soon as the start time point is shifted away from the actual peak. For small infarcts, the number of phases used actually shows a greater influence on the segmentation performance with a modified Dice of 0.5 when using four time points as input to nnU‐Net. Therefore, the initial or baseline phase, which is a window over the 2nd to 4th time point of the CTP series, seems to help the network identify small infarcts. But, using the complete CTP acquisition, as it is done in the experiment with eight equidistant phases as network input, does not outperform the other approaches, proving that simply using more time points is not per se beneficial and, in the context of research question (iii), indicating that careful phase selection can be more effective than using the full perfusion series. Nonetheless, nnU‐Net handles multichannel inputs efficiently—in our experiments, increasing input channels (3–8) did not substantially change training time. While extra channels add CPU/IO work for loading and augmentation, our runs were GPU‐bound, but on hardware with fewer CPU cores, slower storage, or reduced DataLoader parallelism this overhead can dominate, so the cost of using more time points is hardware‐dependent. In general, the proposed time point selection schemes show good performance, especially for infarcts of size 10–70 mL. For comparison, for example, de Vries et al.^24^ reach a Dice score of 0.56 for the segmentation of infarcts (average size 32 mL) using their best model with 16 time points of 2D slices as input.

Overall, this comprehensive study showed the possibilities and limitations of stroke segmentation using different types of CT images and nnU‐Net as segmentation network. While the identification of stroke lesions is still limited in NCCT scans, also due to the development of ischemic changes over time, more advanced scans show a better performance on the task which was expected due to the additional hemodynamic information provided by the use of contrast agent. The best performance can be reached when using CTP scans as model input, for which it is not necessary to utilize the whole series of 3D volumes but rather picking certain time windows and forwarding them concatenated in channel dimension into the network. The mCTA, which can be seen as a “light” version of CTP, also showed good performance but lags behind in the segmentation of small infarcts compared to a perfusion model with four input time points. However, it could be feasible to add the NCCT acquired beforehand as a first phase to the mCTA despite dissimilar image characteristics (e.g., different kernel), as demonstrated by Wang et al.^20^, yielding a scenario that resembles the CTP four time point selection scheme from our experiments. In general, we showed that the mCTA reaches results only slightly below the ones for CTP, highlighting its potential for automatic stroke lesion segmentation. Regarding the transferability of the segmentation models between clinical sites, it could be shown that especially working on NCCT and CTA data benefits from additional fine‐tuning of the models on the new data, while this effect is smaller for mCTA and CTP.

From a clinical perspective, our results indicate that infarct segmentation performance varies substantially across CT image types, highlighting expected volume estimation shifts when advanced scans such as CTP are unavailable. Trials such as DAWN^43^ and DEFUSE‐3^44^ use infarct core thresholds in the range of 50–70 mL to guide thrombectomy eligibility, meaning that even moderate shifts in estimated volume can alter treatment decisions. In our results, these shifts are more clearly reflected in the missing‐versus‐excess volume patterns than in the modified Dice alone, where mCTA and CTP maintain volume estimates much closer to the reference than NCCT and CTA, and therefore align better with clinically relevant decision boundaries. Generally, for translation into clinical practice, we identify two key measures: (i) fine‐tuning pretrained models on small local datasets to reduce domain shifts, and (ii) careful selection and standardization of perfusion time points or mCTA phases to ensure robust and reliable segmentations.

One limitation of this study is the absence of mCTA scans in the data from Center B, preventing direct comparison in the site‐transfer experiments. To address this, we utilized mCTA‐timed extraction of time points from the perfusion series to bridge the gap. However, discrepancies between the image types likely remain, as evidenced by the comparison of mCTA and CTP results from other experiments conducted exclusively on data from Center A. This could be overcome by further experiments on other datasets including mCTA scans. Furthermore, our analysis is restricted to two clinical sites with their specific scanners and protocols, so variability in clinical practice is only partially captured. Potential biases related to local imaging workflows and patient selection may therefore remain and should be investigated in future multicenter validations, including an analysis of how many local cases are required to achieve stable cross‐site adaptation. Generally, the study population is small and especially the limited amount of large infarcts (>70 mL) that made their assessment infeasible is a drawback as their segmentation is relevant for clinical practice and remains to be investigated. Another limitation of the study cohort is that all cases originate from patients imaged for suspected acute ischemic stroke. Combined with the fact that nnU‐Net requires image‐label pairs, this limits systematic inclusion of lesion‐free scans and may bias the model's exposure to normal anatomical variability. Additionally, knowing the stroke onset times would enable even more nuanced evaluated of the results but unfortunately this information was not available for the data used in this study. Moreover, an explicit assessment of inter‐rater variability or annotation reliability (e.g., via consensus labeling or statistical agreement measures) was not conducted in this study. Although the uncertainty‐aware labeling scheme used was designed to reduce subjectivity by leveraging automated perfusion map results, this does not entirely eliminate the potential influence of rater interpretation.

## 5 CONCLUSION

In conclusion, this work presents a broad overview of the use of different CT image types involved in stroke diagnosis and their potential for lesion segmentation with a state‐of‐the‐art DL model. Using CTP showed the best performance on the task with a modified Dice of 0.78 for lesions larger than 10 mL, followed by mCTA, CTA, and NCCT. Fine‐tuning a model on data from a new clinical site before testing has demonstrated to boost performance but depends on the image type and also exhibits limitations. Various strategies how to select subsets of the 4D CTP data for model training were compared and showed good performance overall. Further research is needed particularly on the use of multi‐timepoint imaging, which has been less explored than NCCT and CTA for learning‐based automatic stroke lesion segmentation.

## CONFLICT OF INTEREST STATEMENT

Linda Vorberg, Oliver Taubmann, and Hendrik Ditt are employed by Siemens Healthineers. Savvas Nicolaou is the chief medical officer of SapienSecure.

## Appendix

**TABLE A.1:** Summary of previous deep learning studies on stroke lesion segmentation referenced in Section 1, including imaging modalities, dataset, number of cases, and reported Dice score (if available).

Study	Modality	Dataset	Cases (Train&Val/test)	Dice
El‐Hariri et al. (2022)^11^	NCCT	Internal	438/96	0.40
Hornung et al. (2020)^12^	NCCT	Internal	141/105	0.24‐0.47
Wang et al. (2024)^13^	NCCT	Internal AISD	237/80	0.47 0.62
Lin et al. (2022)^14^	NCCT	Internal	261/‐	0.54
Ni et al. (2022)^15^	NCCT	AISD	345/52	0.52
Mäkelä et al. (2022)^16^	CTA	Internal	50/50	0.61
Giancardo et al. (2023)^17^	CTA	Internal	362/81	0.25
Wang et al. (2021)^20^	NCCT, CTA, (CTA+)	Internal External	345/345 ‐/108	0.38‐0.71 0.32‐0.68
Amador et al. (2022)^21^	CTP	Internal	‐/147	0.45
Mittermeier et al. (2022)^22^	CTP	Internal	217/23	—
Robben et al. (2020)^23^	CTP	MR CLEAN	188/188	0.48
de Vries et al. (2023)^24^	CTP	ISLES 2018	94/62	0.56
Tomasetti et al. (2023)^31^	CTP	Internal	119/33	0.23
Clerigues et al. (2019)^25^	CTP (maps)	ISLES 2018	94/62	0.49
Soltanpour et al. (2019)^26^	CTP (maps)	ISLES 2018	94/62	0.71
Werdiger et al. (2023)^27^	CTP (maps)	Internal	86/22	0.38
Soltanpour et al. (2021)^28^	CTP (maps)	ISLES 2018	94/62	0.68
Bhurwani et al. (2023)^29^	CTP (maps)	Internal	119/119	0.64
Raju et al. (2024)^30^	CTP (maps)	ISLES 2018	63/40	0.58

**TABLE A.2:** Pairwise Wilcoxon signed‐rank tests for modality comparison.

Modality 1	Modality 2	Wilcoxon p	Bonferroni p	Significant
NCCT	CTA	0.003	0.018	Yes
NCCT	mCTA	5.06$\cdot 10^{-7}$	3.03$\cdot 10^{-6}$	Yes
NCCT	CTP	5.23$\cdot 10^{-7}$	3.14$\cdot 10^{-6}$	Yes
CTA	mCTA	2.47$\cdot 10^{-6}$	1.48$\cdot 10^{-5}$	Yes
CTA	CTP	1.45$\cdot 10^{-6}$	8.72$\cdot 10^{-6}$	Yes
mCTA	CTP	0.787	1.0	No

Significance for Bonferroni p<0.05.
